# A Geovisual Analytic Approach to Understanding Geo-Social Relationships in the International Trade Network

**DOI:** 10.1371/journal.pone.0088666

**Published:** 2014-02-18

**Authors:** Wei Luo, Peifeng Yin, Qian Di, Frank Hardisty, Alan M. MacEachren

**Affiliations:** 1 GeoVISTA Center, Department of Geography, Pennsylvania State University, University Park, Pennsylvania, United States of America; 2 PDA Group, Department of Computer Science & Engineering, Pennsylvania State University, University Park, Pennsylvania, United States of America; 3 Department of Epidemiology, Harvard School of Public Health, Boston, Massachusetts, United States of America; University of Gävle, Sweden

## Abstract

The world has become a complex set of geo-social systems interconnected by networks, including transportation networks, telecommunications, and the internet. Understanding the interactions between spatial and social relationships within such geo-social systems is a challenge. This research aims to address this challenge through the framework of geovisual analytics. We present the GeoSocialApp which implements traditional network analysis methods in the context of explicitly spatial and social representations. We then apply it to an exploration of international trade networks in terms of the complex interactions between spatial and social relationships. This exploration using the GeoSocialApp helps us develop a two-part hypothesis: international trade network clusters with structural equivalence are strongly ‘balkanized’ (fragmented) according to the geography of trading partners, and the geographical distance weighted by population within each network cluster has a positive relationship with the development level of countries. In addition to demonstrating the potential of visual analytics to provide insight concerning complex geo-social relationships at a global scale, the research also addresses the challenge of validating insights derived through interactive geovisual analytics. We develop two indicators to quantify the observed patterns, and then use a Monte-Carlo approach to support the hypothesis developed above.

## Introduction

The world has become an increasingly interconnected system with multi-scale geographically embedded networks (i.e., transportation, internet). Spatial analysis aims to understand such systems in terms of spatial patterns, relationships, processes, and change within and among geographical spaces [1]. Social network analysis has been used to understand how systems emerge through the interaction of individual agents (i.e., humans, companies). Both approaches have advantages and limitations as methods through which to understand the complex geo-social interconnected world. Many geo-social interconnected systems mainly grow from the bottom-up, but traditional spatial analysis is a top-down approach that cannot deal with the evolution of the systems over space and time [2], [3]. Social network analysis, a bottom-up approach, can link individual-level behaviors and interactions to the emergence of social phenomena [4], but the approach typically ignores geographical constraints [5]. An effective integration of both approaches has the potential to aid understanding of geo-social systems from a more comprehensive perspective. For example, the integration of spatial consideration into a social network approach enables understanding of why and how an air-borne disease diffuses within an urban area in a manner that can generate disease hot spots as well as cold spots [6]. The integration of spatial analysis and social network analysis has the potential to link individual-level behaviors and interactions (i.e., human, vehicle, organization) to understand urban sprawl over space and time [4]. Although spatial analysis and social network analysis have the potential to complement each other, the formal integration of two approaches remains relatively underdeveloped in the literature [7].

This paper therefore integrates spatial analysis and social network analysis into a unified framework through a geovisual analytics approach. Geovisual analytics tools integrate computational methods with interactive visualization, in order to enable insights on large and complex geospatial datasets [8], [9], [10], [11]. Specifically, we present and apply a geovisual analytics tool, GeoSocialApp [12], that consists of three major analytical “spaces” implemented as linked components: a geographic space, a network space, and an attribute space. Each performs a specific task and can coordinate with other components to facilitate a process through which insights are enabled. We illustrate how the GeoSocialApp facilitates development of hypotheses, with the international trade network (ITN) as a case study. The explicit geographical and network representations in the GeoSocialApp facilitate and enable insight in terms of different roles that spatial and social relationships have in the ITN across geographical regions with network hierarchies at different scales. One major goal of geovisual analytics is to develop hypotheses on how space matters based on the patterns identified from geo-spatial data [13]; but the validation of geovisual analytics results is still regarded as a challenge [14]. Here, we propose a Monte-Carlo approach as a statistical validation to support the hypothesis developed through visual-computational exploration of spatial and social interaction in the ITN.

The paper begins below by reviewing the development of geo-social visual analytics methods in geography and network domains (Section 2). We then present an overview of the methods (Section 3) and the international trade network data used in this study (Section 4). The results obtained through applying the methods to the data (Section 4) provide insights on the different roles that spatial and social relationships play in relation to trade across geographical regions (Section 5). We next introduce the Monte-Carlo approach as a statistical validation to support the insights discussed in section 5 (Section 6). Finally, we present conclusions and an outlook for future research (Section 7).

## Literature Review

Current geo-social visual analytics tools can be classified into two major groups: the first group, rooted in geography, focuses on geographical analysis with an implicitly network representation; the second group, rooted in social network science, has an explicitly network representation with geography as a background to visualize the results. This section reviews the geo-social visual analytics tools from geography and social network science domains, and argues for a more balanced approach that emphasizes spatial relationships and social networks simultaneously.

Spatial interactions/flows associated with topics such as human migration and disease transmission are major research domains for integrating network representation into geovisual analytics. For example, Andrienko and Andrienko [15] develop a spatial generalization method to transform trajectories with common origins and destinations into aggregated flows maintaining essential characteristics of the movement between areas. In complementary research, Guo [16] proposes an integrated interactive visualization framework that is applied to county-to-county migration data in the U.S. in order to visualize and discover network structures, multivariate relations, and their geographic patterns simultaneously. Additional relevant research can be found in recent papers by Andrienko et al. [17], Demšar and Virrantaus [18], Guo, Liu and Jin [19], and Wood, Dykes and Slingsby [20].

All of the above studies consider the geo-social processes from a primarily geographical perspective. Spatial interactions/flows in research taking this perspective are typically visualized on maps, which provide important information on spatial context. The observed spatial patterns can be related to the spatial context (e.g., big cities tend to be hotspots for human interaction). The methods for geo-social interaction discussed so far assume that geographic locations define the geo-social process, but new communication and transportation technologies clearly spread social networks beyond traditional geographical constraints (i.e., distance) [21]. Therefore, understanding the social meaning behind the geo-social processes is equally important.

Geo-social visual analytics from a social network science perspective tends to have an explicit network context with an implicitly geographical representation. Ahmed et al. [22] introduce new visual analysis methods with dynamic network views (e.g., wheel layout, radial layout, and hierarchical layout) to explore the 2006 International Federation of Association Football (FIFA) World Cup competition in which countries are clustered based on their geographical locations in the dynamic graph representation. The visual analysis methods allow users to analyze and compare each country's performance within the geo-social context. The explicit network representation and implicitly geographical representation require analysts to relate the explicit network representation to his or her unrepresented geographic background knowledge in the visually interactive process [8]. Thiemann [23] developed the SPaTo Visual Explorer, which implements multiple explicitly geographical and network representations. Using a case study focused on global air flight networks, he illustrates how SPaTo can allow users to develop hypotheses about the interaction between geographical distance and social network distance. For example, they derive evidence showing that geographical proximity of cities corresponds with short social distance among the cities. Beyond the above, four additional research efforts have focused on specific components of methods to involve explicitly geographical representations into a traditional social network approach: 1) spatial point pattern exploration approach (e.g., kernel density) can be used to understand spatial impacts on the development of social networks [24]; 2) spatial autocorrelation coefficient (e.g., Moran's I) has been applied to social networks to measure the statistical similarity of individuals [25]; 3) explicitly spatial representations facilitate practical implementation of decision-making in certain social network application domains (e.g., infectious disease control) [26]; and 4) certain geo-social systems (e.g., human migration, international trade network) can be better understood or predicted through mathematical models considering physical and social space [27], [28].

As discussed above, understanding geo-social systems requires consideration of both geographical relationships and social network relationships. Therefore, it is necessary to involve explicitly geographical and social network representations. Andris [29] lists five benefits to having an explicit network representation within a geo-spatial framework: 1) the group of connected geographical regions can be studied as a unit with social closeness based on a network community detection approach; 2) the social power of places can be represented by node measures (i.e., degree, betweenness); 3) the social role of interconnected places over the whole system can be represented by network system measures (i.e., degree distribution, betweenness distribution); 4) the complex social interaction between places can be understood through adding multiple social flow layers on Geographical Information System (GIS); and 5) the geo-social systems in which spatial closeness and social closeness do not match can be better modeled with an explicit network representation.

The above discussion illustrates that there is the lack of explicitly spatial and social network representations in current geovisual analytics and the importance of such representations to understand geo-social systems [30]. It is also still a challenge to statistically support the hypotheses developed through visual exploration [31], particularly the hypotheses directed to geo-social interaction. To fill the gap, this paper introduces the GeoSocialApp with the 2005 international trade network as a case study to understand the interaction between spatial and social relationships, and introduces the use of a Monte-Carlo approach to validate the hypothesis developed in our geo-social visual exploration.

## Methods

In this paper, we extend and apply the GeoSocialApp, a geovisual analytics tool initially introduced in preliminary form in Luo et al. [12]. The GeoSocialApp implements traditional network analysis methods within the context of an environment that links explicitly spatial and social representations to understand the interaction of spatial and social relationships in the ITN. The GeoSocialApp is an extension of the GeoViz Toolkit (GVT) developed in the GeoVISTA Center at Penn State [32]. The research presented here makes use of the existing choropleth mapping capabilities of GVT to support geographical analysis as well as the component coordination methods that enable dynamic linking and brushing across views, and adds a dendrogram component that supports multiple graph-based views to represent a varying network hierarchy. Details about other GVT components that could be used to extend the analysis presented here can be found in http://www.geovista.psu.edu/GeoSocialApp/ (The source code for the GeoSocialApp is open source under the Library General Public License, version 2 (LGPL 2.0). We plan a public release of a binary version usable by non-programmers in the future).

## GeoSocialApp Components

As noted above, we use two components in the GeoSocialApp for this study: a dendrogram view and a choropleth view. The dendrogram view implements the convergence of the iterated correlations (CONCOR) algorithm [33], [34] to group nodes with equivalent positions in a single network or multiple social networks together. Equivalent positions refer to collections of actors that have similar ties to and from all other actors in the network. The implication of actors having equivalent positions is that they play similar social roles in a relational network. We can describe the relational network by an adjacency matrix A, which can generate a position similarity matrix R to measure the equivalent positions, whose element value r_ij_ is defined as:
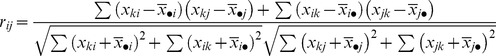
(1)where 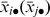
is the mean of the values in row i (j) of the matrix A and 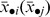
is the mean of the values in column i (j) of the matrix A. At the initial level of analysis, CONCOR performs the above equation calculations iteratively on the position similarity matrix R until all values converge to either 1 or −1, resulting in all nodes being grouped into one of two categories. Two groups can be too generalized for some studies, so hierarchical structures can be achieved by running CONCOR on each subgroup. In this way, CONCOR can continue to split nodes into successively smaller groups: two become four, four become eight, and so on. Although this algorithm was developed originally for application to social networks of individuals, it has been demonstrated to be an effective method to empirically locate structural positions in terms of the ITN [12], [35].

Equivalent positions in terms of the ITN refer to collections of countries that have a similar import and export trade relationships with all other countries [36]. The implication of countries having equivalent positions is that they play similar social roles in the ITN. According to world system theory, the economic development of different countries is affected by their structural positions: core, semi-periphery, and periphery through unequal economic exchanges among them [37]. Core countries focus on capital-intensive production, periphery countries provide low-skill labor and raw materials, and semi-periphery countries are the industrializing countries positioned between the periphery and core countries. The CONCOR algorithm can classify the ITN into these three structural equivalence positions [38], [39].

**Figure 1:**
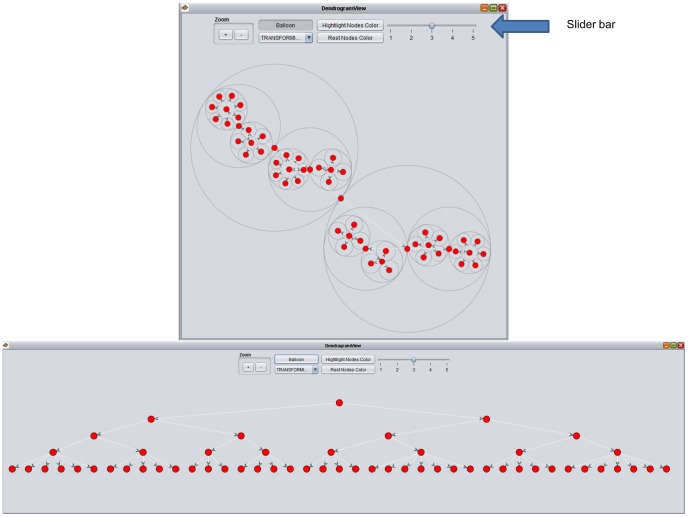
Dendrogram View. Two layouts to visualize the hierarchical structure of CONCOR results: the left one is a tree layout and the right one is a radial layout. Slider bar is used to control the level of CONCOR results.

A tree layout and a radial layout are implemented in the dendrogram view to visualize the hierarchical structure of CONCOR results (Figure 1). The tree layout organizes the graph in a hierarchical way by placing child nodes under their common ancestors. An informationally equivalent radial view can be transformed from the tree by putting child nodes in the enclosing circle of their common ancestors [40], [41]. The dendrogram view in the GeoSocialApp also provides a slider to control the hierarchical level of CONCOR results.

**Figure 2:**
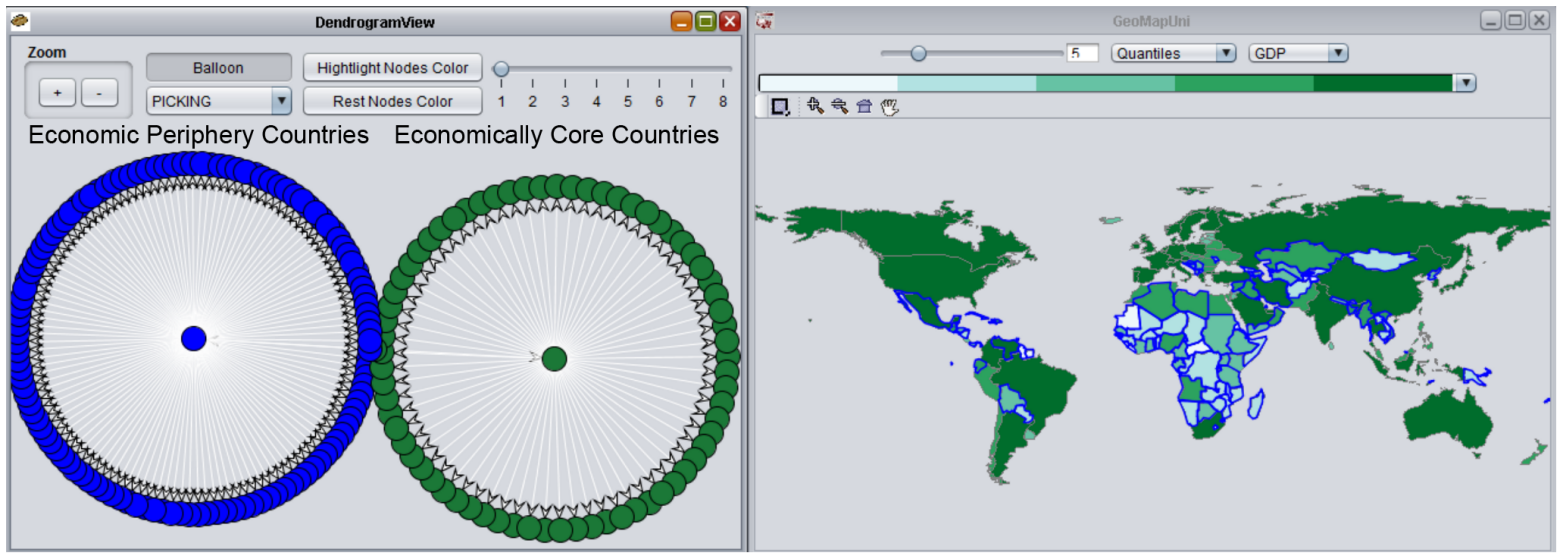
Dendrogram view and choropleth map view. The choropleth map depicts GDP by country. Data are divided into quintiles (5 categories with an equal number of countries in each category) depicted by 5 sequentially ordered shades of green, from low GDP (very light green) to high GDP (very dark green). Each node in the dendrogram view corresponds to one country in the choropleth map view (The highlighted nodes in blue correspond to countries with borders highlighted in blue). The first run of CONCOR process reveals two positions in the 2005 ITN.

The dendrogram view of social space is dynamically linked to a choropleth map view used for visual exploration in geographical space. Each node in the dendrogram view corresponds to a geographical unit (i.e., states, countries) in the choropleth map. The choropleth map allows users to choose the number of classes, the classification method (i.e., equal intervals, quantiles), the variable to display, and the ColorBrewer palette [42] for color selection. Thus, the linked dendrogram and map views allow exploration of social positions and social groups and their corresponding spatial positions and spatial groups simultaneously. With the hierarchical level control in the dendrogram view, the linked views further support the explicit exploration of interaction between social space and geographical space and its impact on outcomes of interest at different network hierarchy (Figure 2). This capability will be illustrated in the case study presented below, after the data used in that case study are first described.

## Data

Our analysis of the interaction between spatial and social relationships in the ITN is based upon import and export data among 192 countries in 2005. These data were extracted from the CorrelatesOfWar (COW) Database and include volume of imports and exports in current U.S. dollars [43]. We convert the 2005 ITN data into a directed network in which countries are the nodes of the network and an import/export trading relationship is represented by a link between two countries. We then organize the data into a binary matrix form to fit the CONCOR algorithm with columns as exporting countries and rows as importing countries. As an illustration, Table 1 is the original import and export data among sample countries in 2005, and Table 2 is the binary matrix for the first 10 countries in our data; “1” represents presence of import/export trade between countries, “0” represents no trade. A binary matrix is used rather than a weighted matrix for twofold reasons: one basic idea of the CONCOR algorithm is that the primary indicator of a relationship is the absence of links between individuals rather than the occurrence of the links [44]; given this idea, the past research in international trade has typically used the binary matrix with the CONCOR algorithm to identify three structural equivalence positions: core, semi-periphery, and periphery [38], [39], [45].

**Table 1:** Imports-exports relationship among partial countries in 2005.

year	importer1	importer2	flow1	flow2
2005	United States of America	Canada	291944	195151
2005	United States of America	Bahamas	726.3	1945.79
2005	United States of America	Cuba	0	397.87
2005	United States of America	Haiti	458.5	756.91
2005	United States of America	Dominican Republic	4721.4	5179.24
2005	United States of America	Jamaica	410.9	1962.2
2005	United States of America	Trinidad and Tobago	8342.2	1583.01
2005	United States of America	Barbados	33.4	595.28
2005	United States of America	Dominica	3.8	67.43

Flow1 means imports of importer1 from importer2 in current US millions of dollars, and flow2 means imports of importer2 from importer1 in current US millions of dollars.

**Table 2:** International trade relationships among partial countries in a binary matrix for 0% threshold in 2005.

	GUATEMALA	BOLIVIA	PARAGUAY	URUGUAY	SURINAME	GAMBIA	MOROCCO	MALI	LIBERIA
GUATEMALA	0	1	1	1	0	0	1	0	0
BOLIVIA	1	0	1	1	0	0	1	0	0
PARAGUAY	1	1	0	1	0	0	1	0	0
URUGUAY	1	1	1	0	1	0	1	0	0
SURINAME	1	0	0	1	0	0	1	1	1
GAMBIA	0	0	0	0	0	0	1	1	1
MOROCCO	1	0	1	1	1	1	0	1	1
MALI	0	0	0	1	0	1	1	0	0
LIBERIA	0	0	1	1	1	0	1	0	0

We use three additional data variables: GDP, population, and geographical distance, to validate the hypothesis developed through visual exploration using the GeoSocialApp. We downloaded 2005 GDP and population data for each country from the World Bank website (http://data.worldbank.org/). We calculated the linear distance between national capitals to measure the geographical distance between countries with ArcGIS. This measure of between-country distance is picked over others (e.g., distance between country centroids, distance between the nearest points of country borders, etc.), because gravity models used in other international trade network studies use the same distance measure [46].

## Results

### Spatial and Social Interaction at the First Level of CONCOR

We use the dendrogram view in the GeoSocialApp to explore Table 2 to identify social relationships among all countries, and the univariate choropleth map to visualize the spatial distribution of GDP for all countries (Figure 2). Comparing the dendrogram view and the map view, and using the dynamic linking between them to explore specific details for individual and groups of countries, can provide insight about spatial and social interactions within the ITN.

Initially, we use the dendrogram view to divide the network data into two groups. After highlighting one group (blue nodes in the dendrogram view and blue outlines in the map view), we find that most countries in the highlighted group are economic periphery countries (i.e., most countries in Central America and Africa) and most countries in the other group are economically core countries (i.e., North America and European Union). The univariate choropleth map depicts GDP for each country. The sequential colors reinforce this classification: economically less-productive countries are indicated by light green, whereas other, more economically productive countries are indicated by dark green. The two classifications identified by CONCOR imply that economically core countries tend to have similar international trade partners, and economic periphery countries tend to have similar trade partners. This study focuses on the interaction between spatial and social relationships in the ITN. At the first level of CONCOR in Figure 2, all countries with close social relationships tend to exhibit spatial proximity.

### Spatial and Social Interaction at the Second Level of CONCOR

**Figure 3:**
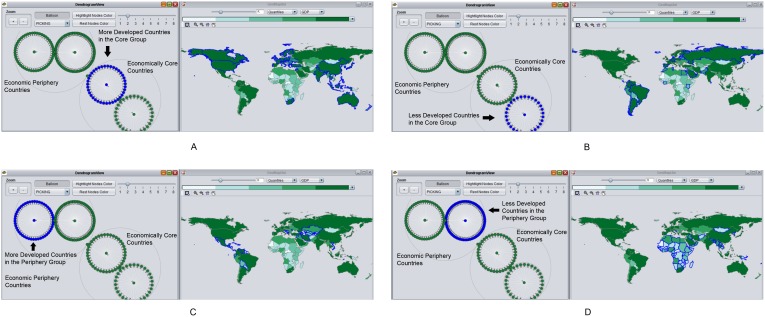
The second run of the CONCOR process subdivides each of the first two groups. Figure 3A: One subgroup of economically core countries; Figure 3B: The other subgroup of economically core countries; Figure 3C: One subgroup of economic periphery countries; Figure 3D: The other subgroup of economic periphery countries.

The second application of CONCOR to the ITN subdivides the first two categories, resulting in a total of four groups as shown by Figure 3 (A list of countries for each group is in File S1.). The core countries and the periphery countries are partitioned into four new geographies, which further indicate a core–periphery arrangement: the mean GDP for each geography is sorted in Table 3. Figure 3A mainly includes more developed countries in the economically core group: North America, most countries in Europe, Australia, South Africa, and economically more-important countries in Asia (i.e., China, India), whereas Figure 3B mainly consists of less developed countries in the economically core groups: Russia, most countries in South America, and a small number of countries in Europe. Figure 3C mainly includes more developed countries in the economic periphery group: Central America, and a few countries from Eurasia (i.e., Vietnam, Iran), whereas Figure 3D mainly consists of the less developed countries in the economic periphery group: countries from Africa and some countries from Asia (e.g., Mongolia). In terms of spatial and social interaction identified by the second level of CONCOR, economically core countries in Figure 3A and Figure 3B (i.e., North America, Europe), as well as more developed periphery countries in Figure 3C exhibit regional patterns (i.e., Central America, Central Asia) that also fall into the same social groups across the globe. It suggests that international trade partners for those countries are related to both spatial proximity and similar economic development level (Figure 3A, 3B, and 3C). Economic periphery countries in Figure 3D have one major cluster (i.e., Africa). Compared to 3A, 3B, and 3C, Figure 3D suggests that spatial proximity has a stronger impact on the least developed countries in terms of international trade partners they have.

**Table 3:** CONCOR group level attribute data.

FigureID	Mean GDP(billions of dollars)	FigureID	Mean GDP(billions of dollars)	Mean Distance(km)	Weighted Distance	Mean GDP(billions of dollars)
3A	912.00	4A	912.00	6664	5.55E+19	912.00
3B		4B	384.00	7146	9.76E+18	384.00
3B	250.00	4C	116.00	8086	5.15E+18	116.00
3C		4D	48.10	3403	1.84E+17	48.10
3C	34.90	4E	21.80	5125	4.67E+17	21.80
3D		4F	13.70	8838	1.19E+18	13.70
3D	12.30	4G	10.90	5833	6.37E+17	10.90

*Mean GDP in 2005 for 4 groups identified at the second level of the CONCOR, mean GDP in 2005, mean distance, weighted distance by population for 7 groups at the third level of the CONCOR.

### Spatial and Social Interaction at the Third Level of CONCOR

**Figure 4:**
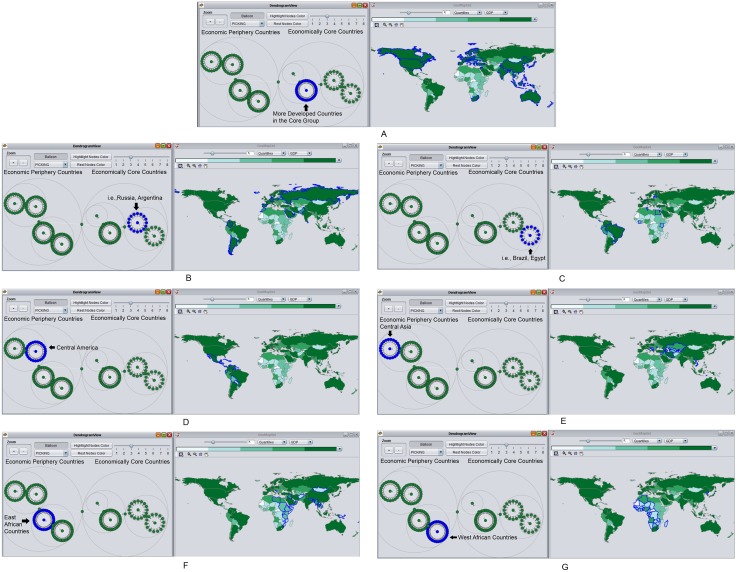
The third run of the CONCOR process continues to subdivide groups. Figure 4A 4B, and 4C belong to the economically core countries, whereas Figure 4D, 4E, 4F, and 4G belong to the economic periphery countries.

The third run of CONCOR applied to the ITN again subdivides the previously identified groups into seven different subgroups (Figure 4) (A list of countries for each group is in File S1.). At this level the geographies are considerably more complex but this research highlights three features. First, only seven new subgroups are identified in this level: CONCOR does not divide countries depicted in Figure 3A any further, resulting in the same group of countries in Figure 4A, because economically core countries in this group have highly similar import and export trade partners. Second, some groups of countries at this level further confirm a core-periphery hierarchical structure in terms of the ITN: the top economically core countries in Figure 4A; a clear distinction between east African countries (the second least developing places) in Figure 4F and west African countries (the least developing regions) in Figure 4G. Third, the role that spatial and social relationships play in terms of the ITN identified by the third level of CONCOR becomes more noticeable. Core countries in Figure 4A, Figure 4B, and Figure 4C have their own distinct geographical regions (i.e., North America, Europe), but social relationships to connect different regions are also strong. Figure 4D and Figure 4E identify two distinct geographical regions (Central America and Central Asia) compared to Figure 3C that put both into the same social group. The distinct geographical regions suggest that spatial constraints are stronger than social connections between the two regions at this network level. Comparing the two distinct geographical regions identified in Figure 4D and Figure 4E to distinct geographical regions (i.e., North America, Europe, and Austria) in Figure 4A suggests that spatial constraints have less impact on economically core countries and more impact on economic periphery countries to determine the international trade partners they have.

## Validation

As outlined above, using an interactive visual approach, we found that developing countries with structural equivalence tend to exhibit a pattern of geographical proximity, and developed countries with structural equivalence tend to exhibit a pattern in which geographical proximity remains a factor, but one that is overcome by some connections to distant places. Based on the patterns, we develop the two-part hypothesis that: international trade network clusters with structural equivalence are strongly ‘balkanized’ (spatially fragmented) according to geography of trading partners, and the geographical distance within each network cluster has a positive relationship with the development level of countries. However, we wish to verify this visual finding with a more robust statistical verification. We have two steps to verify the hypotheses. The first step introduces two indicators (degree of balkanization and Pearson of correlation) to quantify the observed patterns, and the second step uses a Monte-Carlo method to measure the statistical level of the two indicators. It is also important to note that these two linked parts of the analytic process (visual hypothesis generation and confirmatory analysis) provide an iterative means of arriving at stronger conclusions.

### Degree of balkanization

The first part of our hypothesis is that the network cluster with structural equivalence is strongly ‘balkanized’. First, we calculate the average distances between countries that (i) belong to the same cluster and (ii) belong to two distinct clusters. The difference between both distances is a quantification of the degree of balkanization, denoted as *B*. That is to say:
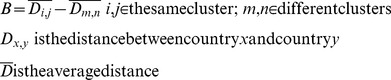



A positive value of B means that countries that belong to the same trade cluster are geographically grouped: the higher the positive value, the higher the degree of balkanization. If B is equal to zero, the countries from the same cluster have no geographic proximity at all and display a random geographic distribution. A negative value of B indicates that countries from the same trade cluster are geographically dispersed. The degree of balkanization of 2005 international trade data set is denoted as 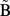, with value of 2774.008 km. The absolute value indicates little about the degree of balkanization unless it is compared to some benchmark. The Monte-Carlo method can provide such a benchmark and produce a statistical significance measure of the absolute result, which we will discuss after describing our approach to measuring the relationship between GDP and distance by network cluster.

### Pearson correlation

We use Pearson correlation [47] to measure the positive relationship between geographical distance within each network cluster and the development level of countries, which is determined by GDP in this paper.
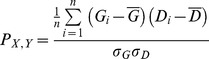



G_i_ is the average GDP of each cluster. D_i_ is the average within-cluster distance of each cluster. σ_G_ is the standard deviation in terms of average GDP of each cluster. σ_D_ is the standard deviation in terms of average within-cluster distance of each cluster. P ranges from −1 to 1. A positive P value implies that there is a positive relationship between geographical distance within each network cluster and GDP. A negative P value implies that geographical distance increases as GDP decreases. If P is around zero, it means that the geographic factor of each network cluster is independent from GDP.

When we calculate the average within-cluster distance, we give more weight to the countries that are more populous by weighting the distance by the population. The reason for this is explained below. The Pearson correlation between the average within-cluster distance without weight and GDP is only 0.13; this does not reflect the strong relationship that is apparent between the two variables as observed visually from the GeoSocialApp. We checked the GeoSocialApp again in order to figure out the reason behind this initial result. We found that simply calculating the average distance between any pair of countries may introduce some noise. For example, island countries in the middle Pacific (Figure 4F) that are far away from any other countries may raise the average within-cluster distance. The cluster in Figure 4F includes mainly developing countries in North Africa and the Mideast, as well as some island countries (e.g. Solomon Islands, Vanuatu). These islands only represent 1.5% of the population and 3.8% of the GDP for the cluster, but increase the within-group distance by 47.71%. Such a dramatic rise of within-group distance makes the distance-GDP nexus indistinct and brings down the Pearson correlation. We test the impact of those islands on the Pearson correlation through removing those islands in Figure 4F, which raises the correlation to 0.36. Given the similar issue existing in some of the other clusters (i.e., Figure 4D, 4E), we weight the distance between all countries proportionally to their population without removing any island countries (Table 3). Following from these preliminary results, we refine our hypothesis into: the geographical distance weighted by population within each network cluster has a positive relationship with the development level of countries. The 2005 international trade data set's Pearson correlation (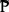) between average GDP per cluster and population weighted within-cluster distance is determined to be 0.97.

### Validation Method

Here, we use a Monte-Carlo method to assess the hypothesis generated from visual-computational exploration. Monte-Carlo methods are a set of mathematical tools that use randomly generated data to evaluate mathematical expressions or to achieve the distribution of some desired variables [48]. Results that are generated from the random inputs serve as benchmarks to determine whether the phenomenon we have observed exhibits a statistically significant difference from that generated by a random process, thus whether the phenomenon is unlikely to have occurred by chance.

**Figure 5:**
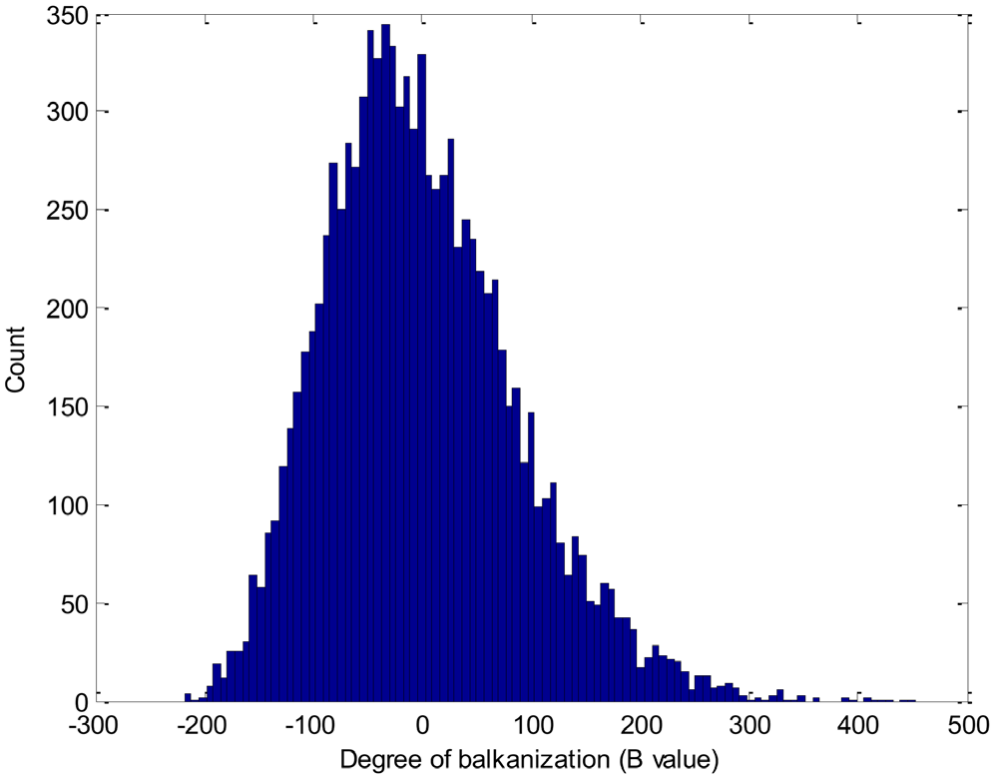
The degree of balkanization of all random trade networks.

To start, we generate 10,000 random international trade networks. The basic idea of this data simulation process is to create trade networks with equal numbers of nodes and links, but to connect the nodes randomly. We keep the number of nodes and links constant to make clustering results from random trade networks comparable to results from the actual ITN data. For each random network, the degree of balkanization *B* and Pearson correlation *P* are calculated after performing the CONCOR algorithm. The 10,000 results offer a numerical approach to calculate the statistical significance of the original degree of balkanization and Pearson correlation by counting the percentage of random networks that have an equal or larger degree of balkanization or Pearson correlation. For the 2005 international trade data set, the degree of balkanization (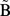) and the statistical significance (p value) of the Pearson correlation (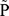) is calculated as follows:
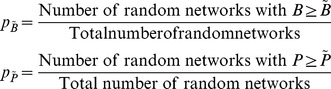



For this analysis, we set the confidence level for p at 0.05. Figure 5 shows the histogram of the degree of balkanization (B) based on all of the random trade networks. This figure shows an imperfect bell-shaped curve, culminating around 0. Its average mean is −0.54, which is very close to 0. An intuitive explanation is that the countries that belong to the same cluster have a random geographic distribution for most random trade networks. The p value of 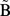 is <0.0001, which means that less than one trade network within every 10,000 random trade networks has a clustering structure that equals or exceeds that of the 2005 international trade network. In other words, the observed high degree of balkanization within the 2005 trade data is unlikely to be a randomly produced result. Thus, the network cluster with structural equivalence exhibits statistically significant geographical clustering.

**Figure 6:**
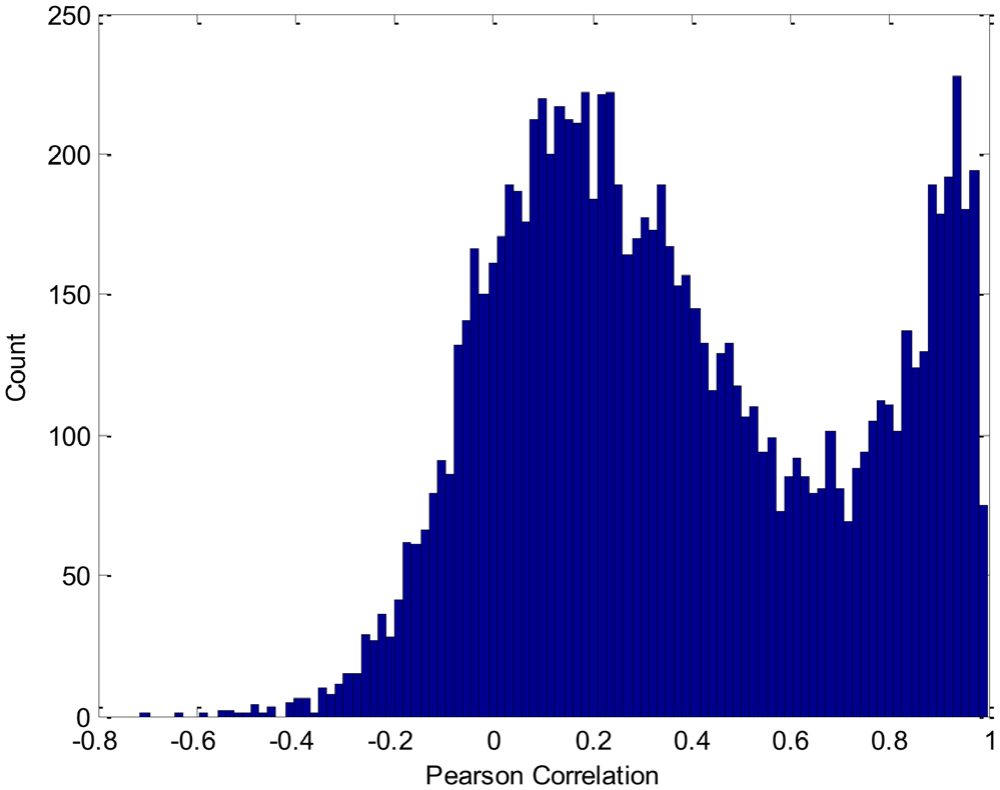
The Pearson correlation values between GDP and weighted within-cluster distance of all random trade networks.

The Pearson correlation values calculated between the average GDP and the weighted within-cluster distance for all random trade networks are displayed in Figure 6. Unlike the previous result in Figure 5, the distribution of Pearson correlation values is irregular with one peak around 0.1 and another mini-peak around 0.9. That the majority of results are associated with the peak around 0.1 can be interpreted to mean that if trade networks were random, the relationship between GDP and the weighted within-cluster distance would be irrelevant or have very weak positive or negative relationship. The bi-modal distribution could be caused by a combination of clusters of countries with similar GDPs and the weighting procedure used. A nearly perfect correspondence between trade clusters and GDP is possible, but if trade links are broken, the patterns rapidly decohere into the default slight positive correlation. Only a small portion of random trade networks exhibit a strong positive relationship between these two variables. The p value is 0.0171, which is significant at 0.05 confidence level. It indicates that less than 2 of every 100 random trade networks display a stronger correlation between GDP and weighted within-cluster distance than found in the actual 2005 ITN data. In other words, the observed strong positive relationship from the visual exploration is unlikely to occur randomly, and the positive relationship between weighted geographical distance within each network cluster and the development level of countries is statistically significant.

### Robustness of the validation method

We use two approaches to test the robustness of the validation results. The first approach is to change the number of runs for each Monte-Carlo validation. The second approach is to create random trade networks with different total connection numbers. For both approaches, we keep the number of nodes constant to make clustering results from random trade networks comparable to original results. If two tests exhibit consistent results with minor fluctuations, such results support that our validation method is robust against these kinds of changes. Similar test approaches have been used in other fields, such as meteorology [49].

**Figure 7:**
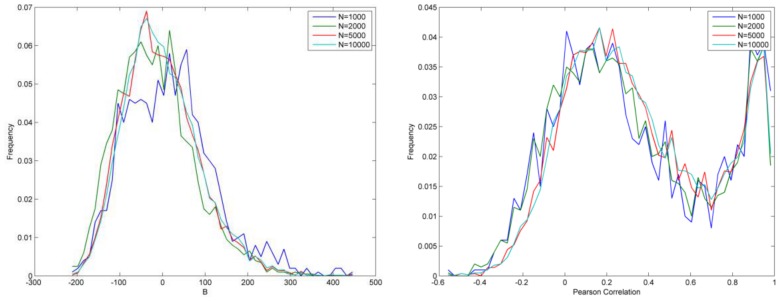
Validation results as a function of number of runs (N).

The first approach examines whether the number of runs in each Monte-Carlo validation influences the final results. If results are robust, validation results will converge as the number of runs increases. Figure 7 displays the results in which the number of runs (N) is 1,000, 2,000, 5,000 and 10,000. When N is small, such as 1,000, the results display some reasonable fluctuations. As the number of runs rises, those results are smoothed and finally converge (as shown by the turquoise line on each plot representing 10,000 runs).

**Figure 8:**
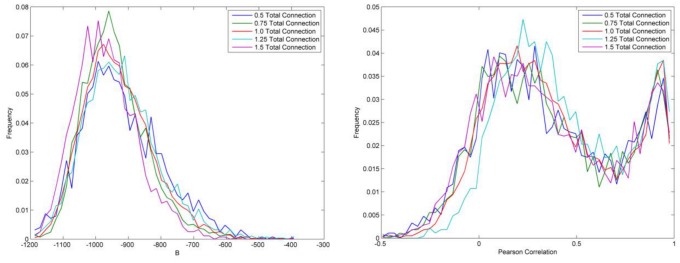
Validation results as a function of total connection numbers.

The second approach uses different numbers of connections among nodes to test the robustness of the validation. We examine the robustness with 50%, 75%, 100%, 150%, and 200% of the original connection number and rerun the validation methods. Figure 8 shows that the distributions of degree of balkanization and Pearson correlation are largely consistent based on the five different scenarios.

This section applies Monte-Carlo methods to validate the hypotheses developed from the GeoSocialApp-based visual-computational exploration of the 2005 ITN. Monte-Carlo simulation produces many randomized pseudo-networks, calculates statistical indicators, and compares the results with those from the original ITN. The results from the 2005 ITN analysis are shown to be statistically significant. In other words, the Monte-Carlo method verifies that the patterns we observe from the GeoSocialApp are unlikely to have resulted from random processes. Moreover, we test the robustness of the validation methods by changing the number of runs and the number of connections. In both scenarios, the Monte-Carlo method produces consistent results, which provides evidence that our validation method is robust.

## Conclusion & Contribution

In this paper, we present the GeoSocialApp, a visual analytics application that supports exploration of the complex interaction between spatial and social network relationships and demonstrate its capabilities by investigating the ITN across geographical regions at different levels of the network hierarchy. The explicit focus of the GeoSocialApp on both geographical and social representations enables a process that generates insight related to the different roles that spatial and social relationships have within the varying network hierarchy levels. To address the network relationships, the GeoSocialApp implements the CONCOR algorithm that has been used in many past studies of the ITN. Although this algorithm has known limitations [50], our focus here is on demonstrating the potential of a geovisual analytics approach that integrates spatial and network analysis methods, not on developing novel methods to measure structural equivalence in networks. In addition, the CONCOR algorithm is still frequently used to measure structural equivalence of the ITN in recent research [28], [45]. Thus, relying on a method with a long history was appropriate. The first run of CONCOR applied to our ITN data suggests a complex interaction between spatial and social relationships for the ITN, but also obscures the separate roles that each relationship has. The second and third run of CONCOR, identifying successively more homogeneous clusters, makes it clear that spatial constraints exist for all groups, but suggests that they are more influential for groups that include economic periphery countries.

Developing hypotheses about phenomena through visual-computational exploration is one major goal of visual analytics; but recent research recognizes that a weakness of many visual analytics methods developed thus far is that they lack mechanisms to validate the hypotheses that are generated [14], [31]. This research develops two indicators to quantitatively assess the patterns identified through visual-computational analysis and then uses a Monte-Carlo method with robustness tests to support our hypothesis with statistical evidence. In addition to using this method to test our hypothesis, we also use the feedback of our first statistical analysis, as discussed in the validation section, to refine our hypotheses. We propose that the approach outlined here may open a new research direction to support iterative hypothesis development, testing and refinement through combined visual-computational exploration and statistical validation.

A future goal for the GeoSocialApp specifically is to integrate this validation method directly within the tools. Monte-Carlo methods are suitable to validate the statistical significance of patterns identified through visual analytics for two reasons: a) patterns revealed through visual analytics tend to be complex and at the same time knowledge about their statistical distributions is absent in most situations; and b) one goal of Monte-Carlo methods is to achieve the distribution of some desired variables with randomly generated data [48]. To effectively integrate Monte-Carlo methods into the visual analytics tools, there are two major challenges: a) how to generate random data to provide baseline distributions based on different applications; and b) Monte-Carlo methods are time-consuming processes because they need to generate a sufficiently large number, e.g., 10,000, of new random data and then calculate the distribution of the desired variables. To address the first challenge, one solution is to understand the process of pattern revelation theoretically and mathematically, and to design Monte-Carlo methods accordingly. To address the second challenge, since each Monte-Carlo realization is completely independent, one solution is to design parallel Monte-Carlo methods, and apply them within a parallel computing environment, e.g., cluster computing frameworks [51].

In addition to integrating the validation method within the application, another future goal for the GeoSocialApp is to convey more information with novel visual designs to improve the process of hypothesis generation. For example, in the radial graphical view, more information (e.g., the distance or GDP distribution within each cluster) could have been symbolized. For the map view, one potentially useful addition might be a paired distance histogram (with 5–7 bins of short to long distance) that summarizes the distribution of between country distances for any selected cluster. In this way, more attribute information can be visualized on the map and network views to understand the interaction between geographical space and social network space.

Social network approaches have been widely applied to study the ITN, with a focus on the importance of network positions and relationships [52], [53], [54], [55]. Fagiolo et al. [56] argue that the role of geographical proximity in shaping the structure of the ITN has not been explored, especially across geographical regions. To fill this gap, recent research integrates two important approaches in the study of global trade: social network analysis and the gravity model [28], [57]. The researchers add network parameters into gravity models to represent the impact of the global trade network on bilateral trade, but those models are still not complex enough to consider both relationships across different geographical regions at varying levels. The hypothesis we developed through visual-computational exploration and then assessed through statistical validation can be considered as another effort toward future international trade models that consider more fully the complex geo-social interactions that occur across different geographical regions at varying levels. Our next step will extend our analysis to the temporal domain in order to understand how such geo-social patterns do change over a longer time period (e.g., from 1989 to 2009).

Given that Pearson correlation is sensitive to the sample size, the high correlation of 0.97 between geographic proximity weighted by population and the development level of countries should be interpreted with caution. However, the goal of this paper is not to produce the definitive analysis of the ITN but to demonstrate the value of applying a geovisual analytics approach as a method to account for both geographic and social network factors in complex processes. Application of the visual-computational methods was able to generate hypotheses about the interaction between level of economic development for countries and relative proximity of international trading partners and the statistical analysis (of which the Pearson correlation is a part) was used to provide support for the hypotheses. The positive relations are further validated statistically and robustly through application of a Monte-Carlo method. In future work, we will consider using a Wilcoxon rank sum test [58] and other similar non-parametric methods to complement the results from Pearson correlation for three reasons: Wilcoxon rank sum test works well even if sample size is small; Wilcoxon rank sum test conducts a formal statistical test and computes a p-value, which provides quantitative information in comparison with descriptive methods like Pearson correlation; non-parametric methods have fewer assumptions and are applicable to more general situations.

The combination of spatial and social network context supports exploration of the interaction between these components and consideration of their impact on outcomes of interest [7], but the combination has not received enough attention generally, not just with respect to the ITN. The GeoSocialApp provides generic frameworks to explore any analysis contexts that include spatial and social relationships among geographical regions (e.g., human migrants among different states in the U.S., war conflicts among different countries in the world, vector borne disease propagation, or the impact of social media on behavior in the world). To our knowledge, this is the first tool to allow users to explore the interconnections of spatial and social relationships at a geographical region level.

## Supporting Information

File S1 **A list of all countries with corresponding group IDs at the second level and third level of CONCOR.**
(PDF)
