# Supplementary material for: A Geovisual Analytic Approach to Understanding Geo-Social Relationships in the International Trade Network
Source: PLoS One. 2014 Feb 18;9(2):e88666. doi: 10.1371/journal.pone.0088666 (PMC3928244; doi:10.1371/journal.pone.0088666)
Supplement: File S1 — A list of all countries with corresponding group IDs at the second level and third level of CONCOR. (PDF) [file pone.0088666.s001.pdf]

COUNTRY_NAME	FigureID	FigureID
AUSTRIA	3A	4A
CZECH REPUBLIC	3A	4A
SLOVENIA	3A	4A
HUNGARY	3A	4A
SLOVAKIA	3A	4A
LITHUANIA	3A	4A
BULGARIA	3A	4A
ISRAEL	3A	4A
JORDAN	3A	4A
SINGAPORE	3A	4A
IRELAND	3A	4A
JAPAN	3A	4A
SWEDEN	3A	4A
FINLAND	3A	4A
PHILIPPINES	3A	4A
TURKEY	3A	4A
TAIWAN, PROVINCE OF CHINA	3A	4A
THAILAND	3A	4A
MALAYSIA	3A	4A
GREECE	3A	4A
INDIA	3A	4A
PORTUGAL	3A	4A
SOUTH AFRICA	3A	4A
POLAND	3A	4A
NORWAY	3A	4A
ROMANIA	3A	4A
UKRAINE	3A	4A
KOREA, REPUBLIC OF	3A	4A
NETHERLANDS	3A	4A
AUSTRALIA	3A	4A
NEW ZEALAND	3A	4A
CHINA	3A	4A
SWITZERLAND	3A	4A
INDONESIA	3A	4A
UNITED KINGDOM	3A	4A
UNITED STATES	3A	4A
FRANCE	3A	4A
ITALY	3A	4A
CANADA	3A	4A
MOROCCO	3B	4B
BELARUS	3B	4B
LEBANON	3B	4B
PAKISTAN	3B	4B
ICELAND	3B	4B
SPAIN	3B	4B
SRI LANKA	3B	4B

CHILE	3B	4B
RUSSIAN FEDERATION	3B	4B
ARGENTINA	3B	4B
GERMANY	3B	4B
COLOMBIA	3B	4B
MALTA	3B	4B
SAUDI ARABIA	3B	4B
UNITED ARAB EMIRATES	3B	4B
DENMARK	3B	4B
URUGUAY	3B	4C
COTE D'IVOIRE	3B	4C
BELGIUM	3B	4C
LUXEMBOURG	3B	4C
LATVIA	3B	4C
CYPRUS	3B	4C
ESTONIA	3B	4C
TUNISIA	3B	4C
COSTA RICA	3B	4C
PERU	3B	4C
BRAZIL	3B	4C
KENYA	3B	4C
MAURITIUS	3B	4C
EGYPT	3B	4C
GUATEMALA	3C	4D
BOLIVIA	3C	4D
PARAGUAY	3C	4D
SURINAME	3C	4D
DOMINICA	3C	4D
SAINT LUCIA	3C	4D
BARBADOS	3C	4D
BAHAMAS	3C	4D
HAITI	3C	4D
DOMINICAN REPUBLIC	3C	4D
JAMAICA	3C	4D
TRINIDAD AND TOBAGO	3C	4D
GRENADA	3C	4D
MEXICO	3C	4D
BELIZE	3C	4D
EL SALVADOR	3C	4D
HONDURAS	3C	4D
CUBA	3C	4D
NICARAGUA	3C	4D
ECUADOR	3C	4D
PANAMA	3C	4D
GUYANA	3C	4D
SAINT KITTS AND NEVIS	3C	4D
SAINT VINCENT AND THE GRENADINES	3C	4D

VENEZUELA	3C	4D
AMERICAN SAMOA	3C	4D
ANTIGUA AND BARBUDA	3C	4D
SAN MARINO	3C	4E
YUGOSLAVIA	3C	4E
ALBANIA	3C	4E
MACEDONIA, THE FORMER YUGOSLAV REPUBLIC	3C	4E
MOLDOVA, REPUBLIC OF	3C	4E
SYRIA	3C	4E
GEORGIA	3C	4E
ARMENIA	3C	4E
KAZAKHSTAN	3C	4E
TURKMENISTAN	3C	4E
UZBEKISTAN	3C	4E
TAJIKISTAN	3C	4E
KYRGYZSTAN	3C	4E
AZERBAIJAN	3C	4E
CROATIA	3C	4E
IRAN	3C	4E
VIETNAM	3C	4E
MICRONESIA, FEDERATED STATES OF	3C	4E
MONACO	3C	4E
ANDORRA	3C	4E
LIECHTENSTEIN	3C	4E
EAST TIMOR	3C	4E
MARSHALL ISLANDS	3C	4E
LIBYA, ARAB JAMAHIRIY_	3D	4F
BOSNIA AND HERZEGOVINA	3D	4F
BURUNDI	3D	4F
UGANDA	3D	4F
SWAZILAND	3D	4F
IRAQ	3D	4F
DJIBOUTI	3D	4F
AFGHANISTAN	3D	4F
NEPAL	3D	4F
MONGOLIA	3D	4F
BHUTAN	3D	4F
LAO PEOPLE'S DEMOCRATIC REPUBLIC	3D	4F
BRUNEI DARUSSALAM	3D	4F
BAHRAIN	3D	4F
QATAR	3D	4F
SOLOMON ISLANDS	3D	4F
SUDAN	3D	4F
YEMEN	3D	4F
OMAN	3D	4F
KUWAIT	3D	4F
BANGLADESH	3D	4F

MYANMAR	3D	4F
PAPUA NEW GUINEA	3D	4F
MOZAMBIQUE	3D	4F
TANZANIA	3D	4F
SOMALIA	3D	4F
MALDIVES	3D	4F
CAMBODIA	3D	4F
VANUATU	3D	4F
TONGA	3D	4F
KIRIBATI	3D	4F
FIJI	3D	4F
TUVALU	3D	4F
ETHIOPIA	3D	4F
ERITREA	3D	4F
GAMBIA	3D	4G
MALI	3D	4G
LIBERIA	3D	4G
ALGERIA	3D	4G
BURKINA FASO	3D	4G
GHANA	3D	4G
TOGO	3D	4G
NIGER	3D	4G
BENIN	3D	4G
CHAD	3D	4G
CENTRAL AFRICAN REPUBLIC	3D	4G
LESOTHO	3D	4G
BOTSWANA	3D	4G
ZAMBIA	3D	4G
ZIMBABWE	3D	4G
RWANDA	3D	4G
MALAWI	3D	4G
KOREA, DEMOCRATIC PEOPLE'S REPUBLIC OF	3D	4G
SEYCHELLES	3D	4G
MADAGASCAR	3D	4G
CAPE VERDE	3D	4G
SENEGAL	3D	4G
MAURITANIA	3D	4G
GUINEA-BISSAU	3D	4G
GUINEA	3D	4G
SIERRA LEONE	3D	4G
NIGERIA	3D	4G
SAO TOME AND PRINCIPE	3D	4G
GABON	3D	4G
EQUATORIAL GUINEA	3D	4G
CAMEROON	3D	4G
CONGO	3D	4G
CONGO, THE DEMOCRATIC REPUBLIC OF THE	3D	4G

NAMIBIA	3D	4G
ANGOLA	3D	4G
COMOROS	3D	4G
NAURU	3D	4G
PALAU	3D	4G
